# Close to the Edge: Growth Restrained by the NAD(P)H/ATP Formation Flux Ratio

**DOI:** 10.3389/fmicb.2017.01149

**Published:** 2017-06-22

**Authors:** Ed W. J. van Niel, Basti Bergdahl, Bärbel Hahn-Hägerdal

**Affiliations:** Division of Applied Microbiology, Lund UniversityLund, Sweden

**Keywords:** NADH, NADPH, ATP, *Saccharomyces cerevisiae*, *Lactobacillus reuteri*, redox imbalance, ATP formation flux, NAD(P)H formation flux

## Abstract

Most fermentative microorganisms grow well-under anaerobic conditions managing a balanced redox and appropriate energy metabolism, but a few species do exist in which cells have to cope with inadequate energy recovery or capture and/or redox balancing. Two cases of these species, i.e., the metabolically engineered *Saccharomyces cerevisiae* enabling it to ferment xylose and *Lactobacillus reuteri* fermenting glucose via the phosphoketolase pathway, are here used to introduce a quantification parameter to capture what limits the growth rate of these microorganisms under anaerobic conditions. This dimensionless parameter, the cofactor formation flux ratio (*R*_*J*_), is the ratio between the redox formation flux (J_NADH+NADPH_), and the energy carrier formation flux (J_ATP_), which are mainly connected to the central carbon pathways. Data from metabolic flux analyses performed in previous and present studies were used to estimate the *R*_*J*_-values. Even though both microorganisms possess different central pathways, a similar relationship between *R*_*J*_ and the specific growth rate (μ) was found. Furthermore, for both microorganisms external electron acceptors moderately reduced the *R*_*J*_-value, thereby raising the μ accordingly. Based on the emerging profile of this relationship an interpretation is presented suggesting that this quantitative analysis can be applied beyond the two microbial species experimentally investigated in the current study to provide data for future targeted strain development strategies.

## Introduction

In intracellular metabolism redox reactions stand central. Many of these reactions are mediated by the cofactors NADH, NADPH, and ATP. There are connections between these co-factors through a complex metabolic interplay and the cell seeks to balance their levels to reach homeostasis (e.g., Holm et al., 2010). Even though NADH and NADPH are usually seen as carriers of reducing power, they can also be considered as energy currencies, like ATP (Alberts et al., 2014). Under aerobic and anaerobic respiration the strong reducing power of NAD(P)H can be converted to ATP by means of an electron transport chain and proton motive force (pmf). This is vital because NAD(P)H needs to be constantly reoxidized to ensure maintenance of metabolic fluxes. Under fermentative conditions, NADH is reoxidized via an organic electron acceptor often provided through the glycolysis, such as pyruvate and acetyl-CoA. In the majority of fermentative microorganisms, the redox and energy balances can reach homeostatic consensus. However, there are examples of anaerobic metabolism where redox imbalance is clearly seen to affect cell growth. Two such cases will be described in this paper in greater detail.

The first case is the longstanding quest to enable yeast to ferment pentoses, with the purpose to have a cost-effective conversion into ethanol from lignocellulosic substrates (Sassner et al., 2008; Moysés et al., 2016). Many metabolic engineering attempts have been made to implement an efficient xylose pathway in *Saccharomyces cerevisiae* that was recently summarized in relation to fermentation of industrial raw material (Sànchez Nogué and Karhumaa, 2015). Like in natural xylose-metabolizing yeasts, these engineered strains cannot (properly) grow anaerobically on xylose. Especially, the xylose pathway consisting of two redox-mediated enzymes, i.e., xylose reductase (XR) and xylitol dehydrogenase (XDH), is hampered with a redox imbalance. This is due to XR being predominantly NADPH-dependent while the XDH is exclusively NAD^+^-dependent (Figure S1). The NADPH-dependent XR has been engineered to increase the affinity for NADH, but the introduction of such a variant into *S. cerevisiae* is not sufficient to enable anaerobic growth on xylose as sole carbon source (Bengtsson et al., 2009), thus it required additional metabolic and evolutionary engineering to accomplish that (Runquist et al., 2009, 2010). Similarly, the alternative strategy to implement a fungal xylose isomerase (XI) to avoid the redox problem displayed anaerobic growth of *S. cerevisiae* on xylose only after several rounds of extensive evolutionary engineering (Kuyper et al., 2004; Kikuta, 2013). The nature of the evolved metabolism has to the best of our knowledge not been disclosed in the public domain. A study has been carried out to visualize specifically the underlying problem using the strain C1 that had evolved as a subpopulation in an anaerobic culture on xylose under carbon-limitation pressure (Sonderegger et al., 2004). The main conclusion, based on metabolic flux analysis and acetoin as external NADH sink, was that absence of anaerobic growth was not determined by the redox balance alone, but also by a limited ATP formation flux, and most importantly, that both are interrelated.

The second case is the intriguing natural metabolism of the lactic acid bacterium *Lactobacillus reuteri* displaying a heterofermentative profile. Investigating the reason why *L. reuteri* ATCC 55730, when grown on glucose, produced more lactate than C2-byproducts (acetate and ethanol), it was found that the central carbon metabolism consists of simultaneous operation of the Phosphoketolase pathway (PKP) and the Embden-Meyerhof-Parnas pathway (EMP; Figure S2, Årsköld et al., 2008). The latter functions only at a minor flux (about 30% of the total carbon-flux), which might for a part be due to a crippled EMP: the *pfkA* gene is missing, but instead the cell can express one or both minor PFK genes (*lr0160* and *lr0378*) that are similar to the *pfkB* gene of *Escherichia coli*. In this case, growth on glucose was not limited by the ATP-formation flux, because in the presence of an external electron acceptor, as NADH sink, the ATP-formation flux did not improve, even though the growth rate had increased nearly 2-fold (Årsköld et al., 2008). It was concluded from these two studies that somehow in both microorganisms redox imbalance imposes a higher demand for ATP. The question as to why *L. reuteri* requires to run a dual central carbon pathway instead of the PKP alone remained unanswered.

In the current paper, a direction to a possible answer is formulated. The determinants behind the described anaerobic growth restriction are proposed in the form of kinetics instead of stoichiometry through a relation between the redox formation flux and the energy carrier formation flux. For this, a dimensionless parameter is introduced, i.e., the NAD(P)H/ATP formation flux ratio, and its graphical relationship with the rate of anaerobic growth of *S. cerevisiae* on xylose and *L. reuteri* on glucose is investigated.

## Materials and methods

### Microorganisms and culture media

The growth data of the various *S. cerevisiae* strains were obtained elsewhere (Table 1): strain TMB 3001 is a haploid CEN.PK117 strain harboring the integrated genes *YYL1* and *XYL2* encoding the *Pichia stipitis* xylose reductase and xylitol dehydrogenase, respectively, and the endogenous *XKS* gene encoding xylulokinase (Wahlbom and Hahn-Hägerdal, 2002); strain C1, an evolved mutant of TMB3001 (Sonderegger et al., 2004); strain 3415 harbors the xylose pathway with a mutated *XYL1* (K270R) gene and overexpresses genes encoding the enzymes of the non-oxidative pentose phosphate pathway [transaldolase (*TAL*), transketolase (*TKL*), ribose 5-phosphate ketol-isomerase (*RKI*), and ribulose 5-phosphate epimerase (*RPE*)] (Runquist et al., 2009); and strains TMB 3420 (selected population), TMB 3421 and TMB 3422 harboring mutated *XYL1* (N272D/P275Q) and *XYL1* (N272D), respectively (Runquist et al., 2010).

**Table 1:** **(A)** Cofactor formation flux ratio (*R*_*J*_) and corresponding specific growth rates of *Saccharomyces cerevisiae* strains on xylose; **(B)** Cofactor formation flux ratio and corresponding normalized specific growth rates of *Lactobacillus reuteri* strains on glucose or sucrose; **(C)** Stoichiometry of redox over energy carrier ratios of various catabolic pathways in yeast and mesophilic prokaryotes under fermentative conditions.

**Strain**	***R_*J*_***	**μ_MAX_ (h^−1^)**	**References**
**A**			
TMB 3057	1.85	0.000	Bergdahl et al., 2012
TMB 3001	1.08	0.000	Wahlbom and Hahn-Hägerdal, 2002
C1	1.023	0.014	Sonderegger et al., 2004
C1 (acetoin)	0.965	0.019	Sonderegger et al., 2004
TMB 3415	0.871	0.025	Runquist et al., 2009
TMB 3421	0.869	0.079	Runquist et al., 2010
TMB 3422	0.823	0.053	Runquist et al., 2010
TMB 3420	0.814	0.068	Runquist et al., 2010
**B**			
**On glucose**			
ATCC 55730	1.353	0.556	This study
ATCC 55730	1.29	0.659	This study
DSM 17938	1.185	0.834	This study
DSM 17938	0.805	0.955	This study
**On sucrose**			
ATCC 55730	0.593	1	Årsköld et al., 2008
**C**			
**NAD(P)H/ATP stoichiometry**			
PKP	1.5		
PKP + e^−^ acceptor	1		
EDP	1		
EMP	0.5		
C3-glycolysis	0.5		

For the model plots of the cofactor formation flux ratio and growth rate the details on the cultivation conditions of *S. cerevisiae* TMB 3057 containing the XR/XDH pathway are described by Bergdahl et al. (2012). In short, the dynamics of the concentrations of a number of intracellular metabolites were monitored by LC-MS/MS in a culture in transit from glucose to xylose metabolism, which also includes the purine nucleotides and redox factors.

The data of the non-engineered type strain *L. reuteri* ATCC 55730 growing on sucrose were obtained as described elsewhere (Årsköld et al., 2008) and growth on glucose was acquired in the current study. *L. reuteri* DSM 17938, the cured strain of *L. reuteri* ATCC 55730 (Rosander et al., 2008), was obtained from BioGaia AB, Stockholm, Sweden. Both strains were routinely anaerobically cultured in MRS (Merck, Darmstadt, Germany) at 37°C in 50 mL tubes (Sarstedt, Nümbrecht, Germany) and the cells were spun down at 4,000 g and kept in 1.5 mL cryovials in MRS supplemented with 10% (v/v) glycerol and stored at −80°C. Precultures inoculated from the cryovials were grown 12 h at 37°C in MRS and a 5% (v/v) inoculum was transferred to SD4, i.e., a semi-defined medium (Levander et al., 2001) with the following additions to the medium (g·L^−1^): NaAc 0.5, MnCl_2_·2H_2_O 0.013, L-glutamine 0.20, and glucose 25. Subsequently, an outgrown culture was used as inoculum (5% v/v) to start 1 L batch-mode cultures in a 2-L pH-controlled Biostat A bioreactor (B. Braun Biotech International, Melsungen, Germany) kept thermostatically at 37°C. The pH was kept at 5.5 through automatic titration with 3 M KOH. The stirring speed was set to 100 rpm. Anaerobic conditions were maintained by the production of carbon dioxide in the culture. Sample treatment and analysis was similar as described previously (van Niel et al., 2012).

### Metabolic flux analysis

For both cases, the metabolic flux analyses were done for batch cultures during the exponential growth phase. The metabolic fluxes through the central carbon pathways operating during anaerobic growth on (i) glucose and xylose in *S. cerevisiae* and (ii) glucose and sucrose in *L. reuteri* were estimated based on stoichiometric models as described in Sonderegger et al. (2004) and Årsköld et al. (2008), respectively. For *S. cerevisiae* the metabolic flux model contained eleven intracellular reactions and five fluxes exchanged with the medium (Table S1), including the glycolysis, pentose phosphate pathway, pyruvate metabolism, and citric acid cycle. For *L. reuteri* the metabolic flux model contained four intracellular reactions and four or five extracellular fluxes, including the PKP, the EMP, pyruvate and acetyl-P metabolism, and the reduction of fructose. The specific consumption and specific production of each metabolite (Y) were estimated during the exponential phase of batch cultures (mmol metabolite· g dry weight^−1^). The fluxes (r) (Table S2) were calculated by multiplying the specific production with the maximum specific growth rate: r = μ_MAX_·Y. Both flux models were solved in MATLAB R2012b (Mathworks, USA). For *S. cerevisiae* the ATP formation fluxes were calculated from the fluxes through glyceraldehyde-3-phosphate (GAPDH) to pyruvate pathway and the acetate pathway, and the NAD(P)H formation fluxes were calculated from the fluxes through the xylose pathway, oxidative pentose phosphate pathway, GAPDH, and the acetate pathway. For *L. reuteri* the ATP formation fluxes were calculated from the fluxes to acetate and lactate, whereas the NAD(P)H formation fluxes were calculated from the PKP flux and the flux through GAPDH.

### Model simulations

The flux distribution among intracellular reactions in *S. cerevisiae* was simulated using the COBRA toolbox in MATLAB R2012b (Mathworks, USA). The genome-scale metabolic model iMM904 (Mo et al., 2009) was updated to generate better predictions of flux distributions during anaerobic glucose and xylose fermentation at steady state. The modifications made to the model included inactivation of genes know to be suppressed during anoxic conditions, revision of the biosynthetic pathways of amino acids, and addition of new reactions that describe the need for FADH_2_ during protein folding in the endoplasmic reticulum. The new model was named iBB912 and contained 912 genes that described 1,102 reactions. Simulated data were generated using a modified version of the dFBA function in the COBRA toolbox. The modifications made in the function were added to allow the use of rate equations for glucose and xylose uptake, glycerol production, and an empirical equation describing the relative flux between the XR:NADH and XR:NADPH reactions (Equations 1–4). The parameters in the rate equations (Table S3) were adjusted to give an accurate description of the fermentation profiles of extracellular metabolites (Figures S3A,B) obtained in a previous study of xylose fermentation (Bergdahl et al., 2012). In the final model, the non-growth-associated maintenance (NGAM) and the growth-associated maintenance (GAM) were set to 0.00907 mmol ATP g^−1^ h^−1^ and 53 mmol ATP g^−1^ h^−1^, respectively. The kinetic equations set the constraints of the flux model and fluxes were calculated using flux balance analysis. The calculated fluxes were used to update the extracellular concentrations after 0.25 h and new constraints were calculated using the kinetic equations. After simulating a total of 40 h, all intracellular reactions in which nadh[c], nadph[c], or atp[c] were produced at any time point, were identified. For each of the three metabolites, the total production flux was calculated by adding together the fluxes of the individual reactions at every time point. The calculated production fluxes, i.e., *R*_NADH_, *R*_NADPH_, and *R*_ATP_, were used to calculate the *R*_*J*_ at every time point of the simulation.

(1) $$\begin{array}{l} \text{Glucose uptake rate }(\text{mmol}/\text{g CDW}/\text{h}):v_{glc}=\frac{V_{\text{max}}^{glc}c_{glc}}{K_{m}^{glc}\left(1+\frac{c_{xyl}}{K_{i}^{xyl}}\right)+c_{glc}}\frac{1}{1+\frac{c_{etoh}}{K_{i,etoh}^{glc}}} \end{array}$$

(2) $$\text{Xylose uptake rate }(\text{mmol}/\text{g CDW}/\text{h}):v_{xyl}=\frac{V_{\text{max}}^{xyl}c_{xyl}}{K_{m}^{xyl}\left(1+\frac{c_{glc}}{K_{i}^{glc}}\right)+c_{xyl}}\frac{1}{1+\frac{c_{etoh}}{K_{i,etoh}^{xyl}}}$$

(3) $$\text{Glycerol production rate }(\text{mmol/g CDW/h}):v_{glyc}=\{\begin{array}{ll} Y_{glyc/(glc+xyl)}\left(v_{glc}+v_{xyl}\right), & v_{glc}>0\\ Y_{glyc/xyl}v_{xyl}, & v_{glc}=0 \end{array}$$

(4) $$\text{XR flux ratio }(\%):R_{XR:NADH}\left(t\right)=A+\frac{K-A}{{\left(1+Qe^{B\left(t-M\right)}\right)}^{1/v}}$$

## Results

### Introduction of the NAD(P)H/ATP formation flux ratio

As the previous studies imply, anaerobic growth of both *S. cerevisiae* on xylose and *L. reuteri* on glucose are in one way or another determined by a cellular redox imbalance combined with a limited ATP-formation flux (Sonderegger et al., 2004; Årsköld et al., 2008). It has been argued that these two factors are highly interrelated, hence they should be captured by a parameter that reflects the state of the cell as such. In addition, the specific growth rate should be directly linked to this parameter. Even though the cells undergo a redox burden, a parameter containing any combinations of NAD(P)H or ATP formation and consumption fluxes will not work, since formation and consumption fluxes will cancel each other out on the metabolic map. Moreover, consumption fluxes, especially those for NADPH and ATP are less traceable as many reactions are involved that cannot be satisfactorily quantified individually. What remains are the formation fluxes of NADPH, NADH, and ATP that mainly take place in the central carbon pathways. The two cases in this study, upon which the proposed parameter is based, have both substantial NADPH formation next to NADH formation in the main catabolic pathways. To take account for this, the fluxes of these two redox cofactors are added together to give the overall redox cofactor formation flux [*J*_*NAD*(*P*)*H*_]. With the energy carrier formation flux (*J*_*ATP*_) we propose to express their interrelationship as a dimensionless quantification parameter, i.e., the cofactor formation flux ratio:

(5) $$\begin{array}{r} R_{J}=\;\frac{J_{NAD\left(P\right)H}}{J_{ATP}} \end{array}$$

with both fluxes having the unit mmol·g dry weight^−1^·h^−1^. The relationship of this parameter with the growth rate will now be analyzed for the two cases presented.

### Case 1: anaerobic growth of *S. cerevisiae* on xylose

**Figure 1:**
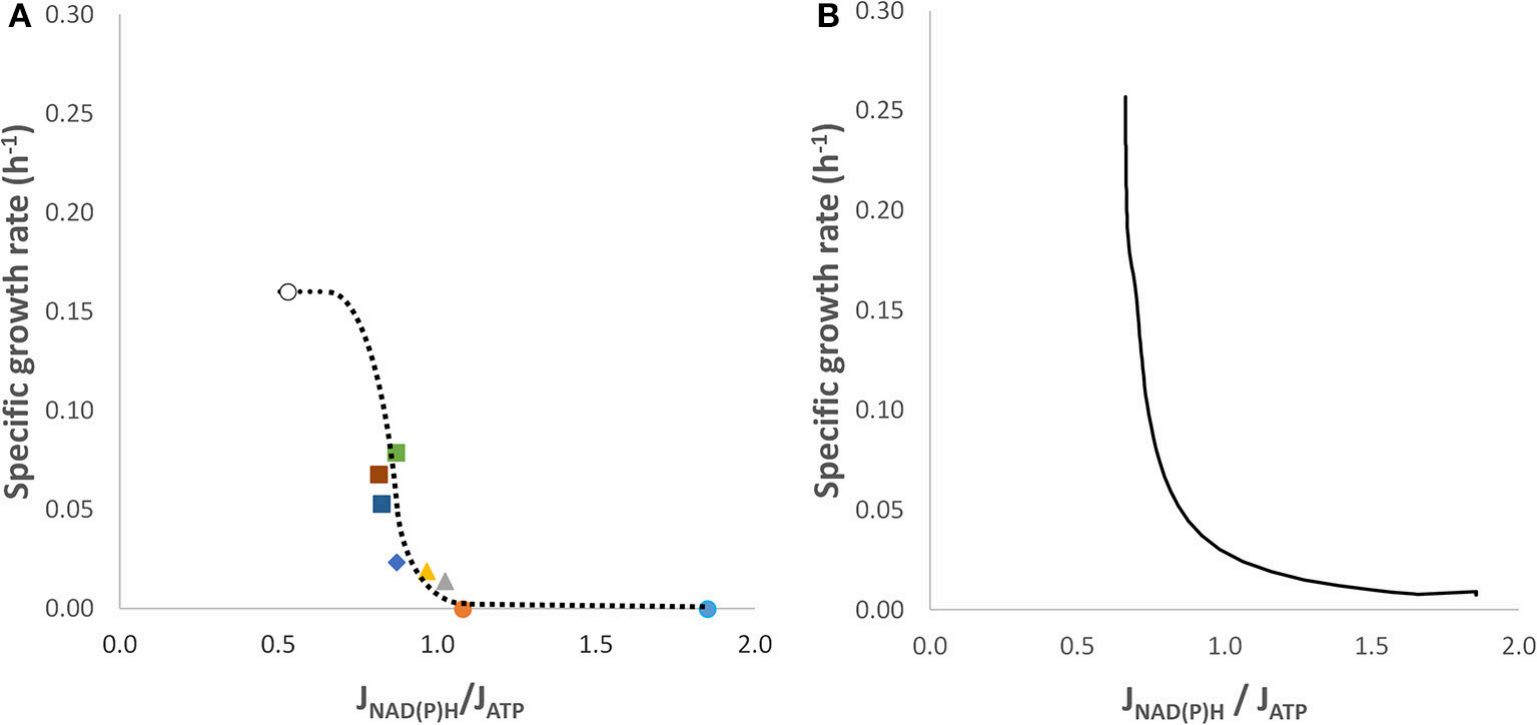
Relationship between the cofactor formation flux ratio and the specific growth rate of various *S. cerevisiae* constructs growing anaerobically on xylose. **(A)** These data points were calculated from various publications (Table 1A; i.e., starting from the right dot to the left): (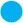) strain TMB3057 (Bergdahl et al., 2012), (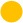) strain TMB 3001, (Wahlbom and Hahn-Hägerdal, 2002); strain C1 without (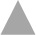) and with acetoin (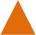), (Sonderegger et al., 2004); strain TMB 3415 (Runquist et al., 2009) (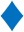); strain TMB 3421 (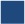), strain TMB 3422 (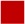), and strain 3420 (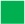) (Runquist et al., 2010). The ideal case (o), i.e., the anaerobically maximum specific growth on xylose has not been established yet, instead a guestimated value is presented here. **(B)** Relationship between the cofactor formation flux ratio and the specific growth rate during the transition from anaerobic glucose/xylose to exclusive xylose metabolism in an anaerobic culture of *S. cerevisiae* TMB3057 as described in Bergdahl et al. (2012).

The long track record of engineering the xylose conversion pathway in *S. cerevisiae* provides a series of strains with successive improvements of anaerobic growth on xylose (Table 1A). Strains TMB 3001 and TMB 3057 fermented xylose without any measurable growth (Eliasson et al., 2000; Bergdahl et al., 2012), whereas strains C1, TMB 3415, TMB 3420, TMB 3421, and TMB 3422 displayed measurable growth rates (Sonderegger et al., 2004; Runquist et al., 2009, 2010). It was observed that by adding an external electron acceptor (acetoin) to cultures of strain C1 the growth rate increased by 36% and the *R*_*J*_*-*value decreased accordingly (Table 1A). Each of these studies contained sufficient data to estimate the metabolic fluxes through the central carbon pathways and allowed to estimate values for *R*_*J*_, revealing that several of them are above 1.0, with strains having a *R*_*J*_-value of ca. 0.81 displaying measurable growth (Table 1A). Unfortunately, the maximum anaerobic specific growth rate on xylose is not known, but a rough value was calculated as follows. Maximum specific growth rates of *S. cerevisiae* on glucose aerobically: anaerobically = 1: 0.66, as estimated from data of Petersson et al. (2006) and Verduyn et al. (1990). Thus in analogy, the anaerobic maximum specific growth on xylose was assumed to be 0.66 × aerobic specific growth rate on xylose (0.21–0.28 h^−1^, Jeppsson et al., 2003) or 0.16 ± 0.033 h^−1^. The corresponding *R*_*J*_-value (0.529) is based on absence of a redox imbalance (no acetate formation, minimum flux through the oxidative PPP and maximum flux to ethanol). In contrast, at high *R*_*J*_-values cellular metabolism lacks capacity for growth. Interestingly, plotting the discrete values of *R*_*J*_ against the corresponding maximum specific growth rate revealed a sigmoidal profile (Figure 1A).

A later study (Bergdahl et al., 2012) aimed to capture the dynamics of intracellular metabolite concentrations in a *S. cerevisiae* TMB 3057 culture shifting its metabolism from glucose to xylose fermentation. The established data set allowed to simulate this transition accurately for biomass production, and glucose and xylose consumption (Figures S3A,B). The model generated a continuous line of the transition of the maximum specific growth rate on glucose to an almost zero growth rate on xylose (Figure S3C) as well as the change in *R*_*J*_ (Figure S3D) and the relationship between *R*_*J*_ and the specific growth rate (Figure 1B). The value of *R*_*J*_ changed from 0.66 (with glucose plus xylose), at which the growth rate was maximal, to 1.85 (only xylose; Table 1) where growth had nearly ceased. During the shift glucose and xylose were taken up and metabolized simultaneously, after which the xylose metabolism became gradually more dominant as the glucose approached complete consumption (at *t* = 21 h, Figure S3B). This simulation illustrates the gradual transition of the *R*_*J*_ from the full growth to the non-growth situation very well. Note that the profiles in Figures 1A,B are very similar, however, the former is based on fermentation xylose alone, whereas in the latter glucose and xylose were converted simultaneously during the shift (compare Figure S3B with Figure S3D).

### Case 2: anaerobic growth of *L. reuteri* on glucose

**Figure 2:**
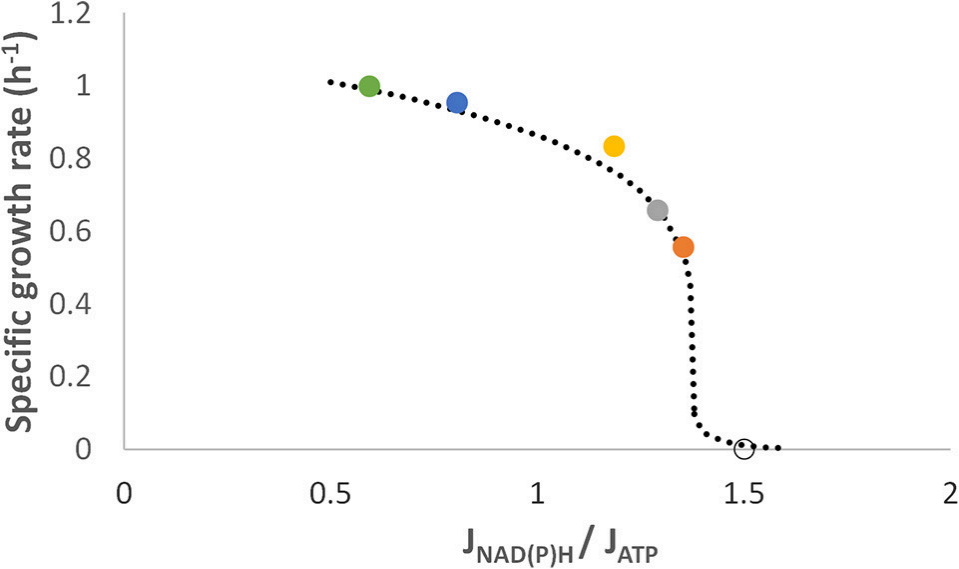
Relationship between the cofactor formation flux ratio and the normalized specific growth rate of various *L. reuteri* strains growing anaerobically. These data points were calculated from (left to right): (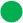) strain ATCC 55730 on sucrose (Årsköld et al., 2008), (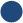, 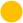) strain DSM 17938 on glucose (this study) and (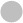, 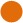) strain ATCC 55730 on glucose (this study). No growth (o) is assumed to be in the case there is zero flux through the EMP (100% flux through the PKP) and thus the *R*_*J*_ is equal to 1.5 (Table 1C).

In this case, several natural strains are involved, each possessing its own maximum specific growth rate. Therefore, the specific growth rate was normalized for construction of the growth vs. *R*_*J*_ plot. Part of the data originated from a *L. reuteri* ATCC 55370 culture on sucrose (Årsköld et al., 2008) and on glucose (this study) and a part from *L. reuteri* DSM 17938 cultures on glucose (this study; Table 1B). For this organism the fructose half of sucrose functions only as an external electron acceptor, therefore higher growth rates are obtained for cultures on sucrose than for cultures on glucose and concomitantly the *R*_*J*_-value declines accordingly, like for *S. cerevisiae* on xylose and acetoin. Plotting the *R*_*J*_ against the corresponding specific growth rate again suggests a sigmoidal profile even though there is a lack of data at the low growth rates (Figure 2). Therefore, we added a theoretical point for the case there is a 100% flux through the PKP, and no flux through the EMP pathway, at which the value of *R*_*J*_ should become 1.5 and our expectation is that the specific growth rate will be close to zero (Figure 2).

## Discussion

In this paper, we introduce a dimensionless parameter to quantify a physiological phenomenon that has been observed in some microorganisms under anaerobic conditions, of which one is the xylose metabolism manufactured in *S. cerevisiae* by metabolic engineering and the other is glucose metabolism in *L. reuteri*. This parameter is a ratio between the formation fluxes of redox carriers and energy carrier and is thus based on the kinetics rather than the stoichiometry of the reactions. This parameter (*R*_*J*_) was logically built on the conclusion of a previous study that a combination of redox imbalance and a limited ATP formation flux forces metabolically active cells of *S. cerevisiae* on xylose to meander close to the edge of propagation (Sonderegger et al., 2004). A sigmoidal correlation between *R*_*J*_ and the specific growth rate was substantiated through plotting new and old data of two cases, i.e., *S. cerevisiae* fermenting xylose and *L. reuteri* fermenting glucose (Figures 1A, 2). The excellent match between the continuous line generated in the simulation of the metabolism of a single strain (Figure 1B) with the profile that emerged from plotting discrete datapoints of various strains (Figure 1A) gives weight to apply *R*_*J*_ for describing the metabolic state of the cell under such confined growth.

In the case of anaerobic cultures of metabolically engineered *S. cerevisiae* strains on xylose, cells are continuously consuming the sugar, thereby maintaining their high energy charge, but still keeping them in a state of restrictive growth (Bergdahl et al., 2012). A perpetual process enrolls in which energy extracted in the fermentation of xylose is used in a futile correction of the redox imbalance, which is similar to the uncoupling of energy metabolism and anion accumulation in cells when exposed to weak acids at low pH (Russell, 1992). The existence of both the redox imbalance and lack of sufficient ATP formation are inherent to the creation of the xylose metabolism in these engineered strains. The moderately mitigation of the redox imbalance through addition of an external electron acceptor increased the growth rate and lowered the *R*_*J*_-value accordingly (Table 1A).

In the case of *L. reuteri* growth on glucose allows a fair growth rate, most probably due to that this microorganism possesses two simultaneously operating central carbon pathways as a way to partially alleviating the redox burden while acquiring an improved ATP formation flux as well. Again as for xylose metabolizing *S. cerevisiae*, this can be further mended by adding an external electron acceptor, leading to both higher growth rate and cell yield (Årsköld et al., 2008): the growth rate on sucrose (the fructose part acted as the external electron acceptor) was 1.82-fold higher than the growth rate on glucose. As expected, the corresponding *R*_*J*_-value for the sucrose-grown cells was seen to decrease substantially (Table 1B). The same can be said for *Leuconostoc oenos* that can use citrate as an electron acceptor. As it relies entirely on the PKP as its central carbon pathway, its growth rate is at the very low, i.e., 0.087 and 0.038 h^−1^ with and without citrate, respectively (Salou et al., 1994). The corresponding *R*_*J*_-values were 1.12 and 1.32, respectively, as estimated from the graphical information given in their paper.

Does this phenomenon relate to growth at near-zero growth rates? There has been a renewed interest in microbial physiology of anaerobically growing microorganisms, both prokaryotes and eukaryotes at near-zero to zero growth rates (Goffin et al., 2010; Boender et al., 2011). One approach is by using rentostats to obtain growth rates as low as 0.0002 h^−1^ for *Lactobacillus plantarum* (Goffin et al., 2010); a second approach is to have cultures growing up in batch mode on a rich medium after which the cells are allowed to enter a prolonged stationary phase in which the quiescent state of *S. cerevisiae* cells is obtained (Gray et al., 2004). In both cases, the cultures started from high growth rates after which they decelerate gradually to the (almost) non-growth state. Monitoring the metabolic activity of *L. plantarum* in the rentostat during the gradual growth rate decline, the cells maintained the same physiology at all growth rates, including the absence of diversion toward higher ATP-yielding pathways (Goffin et al., 2010). On the other hand, *S. cerevisiae* at near-zero growth rates, like stationary phase cells, enter quiescence leading to a changed phenotype related to carbon starvation (Gray et al., 2004; Boender et al., 2011), but growth was never limited by redox or energy fluxes. In other words, in this approach neither redox burdens nor shortages of ATP formation fluxes were created, hence *R*_*J*_-values will remain near 0.5.

The sigmoidal profile of the plot for *S. cerevisiae* (Figure 1) is quite similar to that of *L. reuteri* (Figure 2), even though the approach of the curve is from the “bottom up” concerning the former and from the “top down” for the latter. Interestingly, the profile that emerged from each of the plots reflects a second-order phase transition, which is characterized by the continuous transition shape (Waigh, 2007). As a consequence, there is a critical *R*_*J*_ that is situated at the inflection point of the profile (most visible in Figure 1A) similar to the melting temperature of DNA in the classical GC-content determination. It can be argued that the critical value for prokaryotes is equal to ≈1.0–1.3 and for yeast ≈0.8. The nature of the critical *R*_*J*_ merits further investigation, and might represent the critical point where the redox formation and ATP formation fluxes match beyond which the “restrictive growth mode” dissolves. In addition, the underlying sigmoidal pattern might be related to the spatial-temporal nature of this phenomenon occurring in a molecular crowded cytoplasm that affects diffusion of the cofactors to their targets (Klann and Koeppl, 2012).

An explanation for the summation of the formation fluxes of NADH and NADPH can be either setting the redox potential or their accompanying protons, in other words the J_NAD(P)H_ represents also a proton formation flux (J^H+^). However, the influence of the redox can be questionable. On the one hand, both the NADPH/NADP ratio and the NADH/NAD ratio declined somewhat during the shift from glucose to xylose metabolism in *S. cerevisiae* TMB 3057 (Bergdahl et al., 2012), and on the other hand glutathione with an intracellular concentration of ca. 10 mM (Penninckx, 2002) will be present in a 5-fold higher concentration than the sum of the two redox carriers and thus will dominate the redox potential. A proton formation flux would imply that at high *R*_*J*_-values the cytoplasm might be relatively more acidic. This needs to be verified with the strains mentioned in this study, but the observation of the intracellular pH being lower in xylose-metabolizing than in glucose-metabolizing *Pichia stipitis* and *Candida tropicalis* as measured with ^31^P-NMR (Lohmeier-Vogel et al., 1995, 1996) represents a good indication. Hence, the *R*_*J*_-value might reflect the success of the excretion of protons over the cell membrane as mediated by ATPase. Indeed, under anaerobic conditions, the cytoplasmic pH (pH_C_) is dependent on the level of the H^+^-ATPase activity (Kobayashi, 1985). Addition of external electron acceptors did remedy this burden to a moderate extent for both *S. cerevisiae* and *L. reuteri* (Tables 1A,B). Therefore, with a *R*_*J*_ beyond a critical value the J_ATP_ would be simply too low to cope sufficiently with the demand for the proton excretion flux. In particular, *S. cerevisiae* is severely restricted in its efficiency to remove protons from the cytosol as it costs 1 ATP per excretion of 1 proton (Van Leeuwen et al., 1992).

Orij et al. (2012) demonstrated that the pH_C_ directly controls the growth rate in *S. cerevisiae*, with a reduction in pH_C_ leading to lower growth rates. Furthermore, transient changes in the pH_C_ function as signals in yeasts for various processes in the cell, including positive feedback to initiate glucose metabolism, cell cycle progression, apoptosis, and morphological changes (Orij et al., 2011; Kane, 2016). Intracellar pH does not seem to be having a signaling role in bacteria, but pH homeostasis is controlled by proton-ATPases and Na^+^/H^+^ and K^+^/H^+^ antiporters (see e.g., Krulwich et al., 2011). Yet, it remains to be investigated how the cofactor formation flux ratio is involved in any cytosolic acidification as the latter is imposed by several other cellular processes and environmental conditions (Kane, 2016).

*L. reuteri* maintains high growth rates at higher *R*_*J*_-values than *S. cerevisiae* (Figure 1 vs. Figure 2). This is probably due to that in the eukaryote ATP is converted in more cellular processes than in the prokaryote, especially when the organelles are taken into account. This also includes mitochondria, as under anaerobic conditions these organelles rely on cytosolic ATP to maintain a proper proton motive force, which may amount to at least 30 nmol ATP·mg protein^−1^·min^−1^ (Venard et al., 2003).

The cofactor formation ratio can also be used to reevaluate central carbon metabolisms in prokaryotes (Tables 1B,C). To compare the EMP with the PKP and the Entner-Doudoroff pathway (EDP) as isolated pathways in the absence of any metabolic flux analyses, we can use the stoichiometric ratio of the redox formation and energy carrier formation [NAD(P)H/ATP, mol·mol^−1^]. With respect to this ratio, it is clear that the EMP possesses the lowest *R*_*J*_*-*value compared to the EDP and PKP with or without an electron acceptor (Table 1C). This pathway operates ideally under fermentative conditions, leading to relatively high growth rates and biomass yields. Therefore, with a formation flux ratio of 0.5 this catabolic pathway can be used as a benchmark. It is the lower half of the EMP, the C3-branch, which determines its formation flux ratio (Table 1C). Interestingly, this C3-branch is shared with all the other catabolic pathways that include more oxidation steps than the EMP and thus raises the redox formation flux (Table 1C). This simple comparison makes clear that the PKP owns the highest *R*_*J*_-value and it can be argued whether microorganisms possessing only this central carbon pathway will grow under anaerobic conditions in the absence of an appropriate electron acceptor. That might give the answer to the question why *L. reuteri* possesses a “crippled” EMP in addition to the PKP: to enable growth under anaerobic conditions. The same can be considered for anaerobic microorganisms having only the EDP. This topic has been addressed recently (Flamholz et al., 2013), and indeed these metabolic types are hardly present among anaerobes.

One notable exception is *Zymomonas mobilis* that anaerobically grows best in the presence of high glucose concentrations (>0.5 M). It earns very low biomass yields due to a net yield of 1 mol ATP·mol glucose^−1^ (Fuhrer et al., 2005). *Z. mobilis* is able to do so because of having a metabolic highway from glucose to ethanol comprising 50% of the total cell protein and other physiological adjustments, allowing for exceptionally high metabolic fluxes to ethanol and only minor fluxes to biosynthesis (Sprenger, 1996; Fuhrer et al., 2005; Seo et al., 2005; Lee et al., 2010; Widiastuti et al., 2010). Thus, the anaerobically operating EDP comes with a price: minimum biomass yields are acquired but its existence is restricted to environments containing high sugar concentrations. Another exception is *E. coli* that is only able to grow anaerobically on xylose in the presence of pyruvate formate lyase (PFL; Hasona et al., 2004). Under that condition, equal amounts of ethanol and acetate corresponds to a *R*_*J*_ of 1.11; a Δ*pfl* mutant failed to grow, but then the value for *R*_*J*_ would have been a fatally high 2.5.

A step toward answering the long-standing question why certain types of metabolic pathways do not guarantee growth under anaerobic conditions is proposed here via introduction of a quantification parameter, *R*_*J*_, based on kinetics. The current analysis of the available data reveals a relationship between this new dimensionless parameter and the specific growth rate. In what mechanistic way, the cofactor formation flux ratio is connected to acidification of the cytosol merits specific investigation. The value of this parameter implies how far the metabolism is from the critical situation beyond which unrestricted growth is allowed. Therefore, *R*_*J*_ can be used as a novel analytical tool for targeted design of new strains, and is easily implemented in metabolic flux model simulations.

## Author contributions

Ev conceived and designed part of the experiments and drafted the manuscript with BH. BB performed the model simulations. All authors critically read, contributed to, approved the final version of the manuscript, and agreed to be accountable for all parts of the work.

### Conflict of interest statement

The authors declare that the research was conducted in the absence of any commercial or financial relationships that could be construed as a potential conflict of interest.

## Supplementary material

Figure S1 Central metabolic pathways in *S. cerevisiae* capable of fermenting glucose and xylose. The pathways include the Embden-Meyerhof-Parnas pathway, pentose phosphate pathway, the engineered xylose pathway, and the pathways leading to acetate and ethanol. FBP, fructose bisphosphate; DHAP, dihydroxyacetone phosphate; GAP, glyceraldehyde 3-phosphate; PYR, pyruvate; R5P, ribose 5-phosphate; S7P, sedoheptulose 7-phosphate; E4P, erythrose 4-phosphate.

Figure S2 Central metabolic pathways in the heterofermentative *L. reuteri*. The central pathways consists of the phosphoketolase pathway (from glucose 6-phosphate to acetyl phosphate (AcP) and glyceraldehyde 3-phosphate (GAP) and the Embden-Meyerhof-Parnas pathway. Either glucose or sucrose is used as substrate, in which the latter is split into glucose and fructose. The latter is used as electron acceptor being reduced to mannitol and the former enters the central carbon pathways via glucose 1-phosphate. F6P, fructose 6-phosphate; FBP, fructose bisphosphate; DHAP, dihydroxyacetone phosphate; AcCoA, Acetyl-CoA; PYR, pyruvate.

Figure S3 Transition profiles of the metabolism of an anaerobic culture of *S. cerevisiae* TMB 3057 from glucose to xylose in batch cultures as described in Bergdahl et al. (2012). The symbols represent the measured data points and the lines are generated by the simulation model. **(A)** Biomass production. **(B)** Glucose (o) and xylose (□) consumption. **(C)** Specific growth rate. **(D)** Change of the cofactor formation flux ratio (*R*_*J*_) from simultaneous glucose and xylose metabolism to exclusive xylose metabolism.

Table S1 Metabolite consumption and production rates for *S. cerevisiae* during exponential growth on xylose. Symbols: μ, specific growth rate (h^−1^); r, specific consumption/production rates (mmol·g DW^−1^·h^−1^); r_S_, specific substrate consumption rate; r_Xol_, specific xylitol production rate; r_G_, specific glycerol production rate; r_A_, specific acetate production rate; r_E_, specific ethanol production rate.

Table S2 Metabolite consumption and production rates for *L. reuteri* during exponential growth on glucose or sucrose. Symbols: μ, non-normalized specific growth rate (h^−1^); r, specific consumption/production rates (mmol·g DW^−1^·h^−1^); r_S_, specific substrate consumption rate; r_L_, specific lactate production rate; r_E_, specific ethanol production rate; r_A_, specific acetate production rate.

Table S3 Parameter values of the kinetic equations to estimate the fermentation profiles of the external fluxes of *S. cerevisiae* strain 3057. Symbols: $V_{max}^{glc}$, the maximum specific glucose conversion rate; $K_{m}^{glc}$, the affinity constant for glucose; $K_{i}^{xyl}$, the xylose inhibition constant for glucose conversion; $K_{i,etoh}^{glc}$, the ethanol inhibition constant for glucose conversion; $V_{max}^{xyl}$, the maximum specific xylose conversion rate; $K_{m}^{xyl}$, the affinity constant for xylose; $K_{i}^{glc}$, the glucose inhibition constant for xylose conversion; $K_{i,etoh}^{xyl}$, the ethanol inhibition constant for xylose conversion; Y_glyc/(glc+xyl)_, the glycerol yield on glucose and xylose; Y_glyc/xyl_, the glycerol yield on xylose. The ratio between the XR:NADH and XR:NADPH reactions was described by a generalized logistic function with the following parameters: A, the lower asymptote; K, the upper asymptote; Q, affects the value of the ratio at time, *t* = 0; B, the growth rate; M, sets the starting time; ν, affects near which asymptote maximum growth occurs.
