# Supplementary figures and images for: Close to the Edge: Growth Restrained by the NAD(P)H/ATP Formation Flux Ratio
Source: Front Microbiol. 2017 Jun 22;8:1149. doi: 10.3389/fmicb.2017.01149 (PMC5479917; doi:10.3389/fmicb.2017.01149)

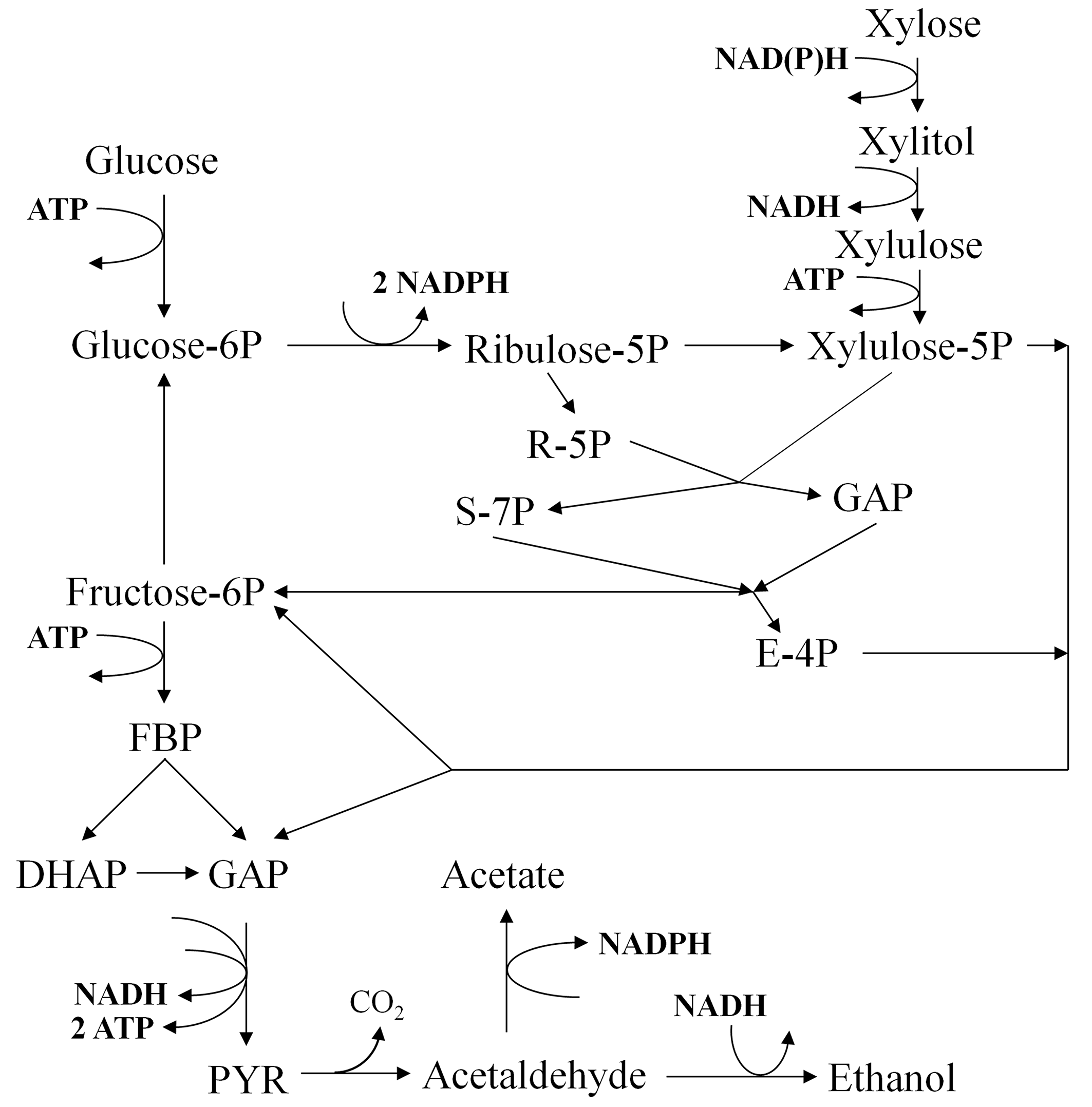

Supplement: Figure S1 — Central metabolic pathways in S. cerevisiae capable of fermenting glucose and xylose. The pathways include the Embden-Meyerhof-Parnas pathway, pentose phosphate pathway, the engineered xylose pathway, and the pathways leading to acetate and ethanol. FBP, fructose bisphosphate; DHAP, dihydroxyacetone phosphate; GAP, glyceraldehyde 3-phosphate; PYR, pyruvate; R5P, ribose 5-phosphate; S7P, sedoheptulose 7-phosphate; E4P, erythrose 4-phosphate. [file Image1.TIF]

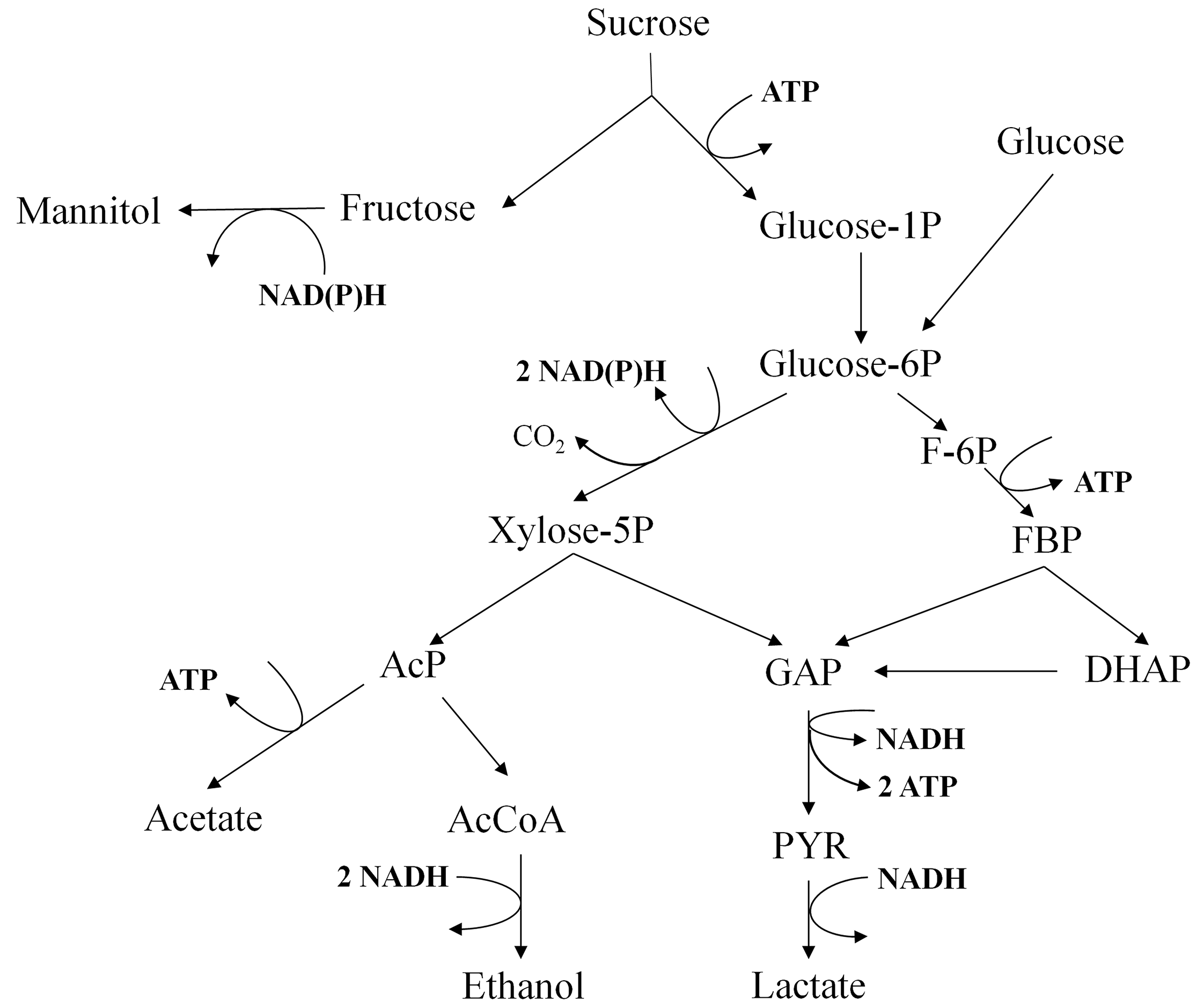

Supplement: Figure S2 — Central metabolic pathways in the heterofermentative L. reuteri. The central pathways consists of the phosphoketolase pathway (from glucose 6-phosphate to acetyl phosphate (AcP) and glyceraldehyde 3-phosphate (GAP) and the Embden-Meyerhof-Parnas pathway. Either glucose or sucrose is used as substrate, in which the latter is split into glucose and fructose. The latter is used as electron acceptor being reduced to mannitol and the former enters the central carbon pathways via glucose 1-phosphate. F6P, fructose 6-phosphate; FBP, fructose bisphosphate; DHAP, dihydroxyacetone phosphate; AcCoA, Acetyl-CoA; PYR, pyruvate. [file Image2.TIF]

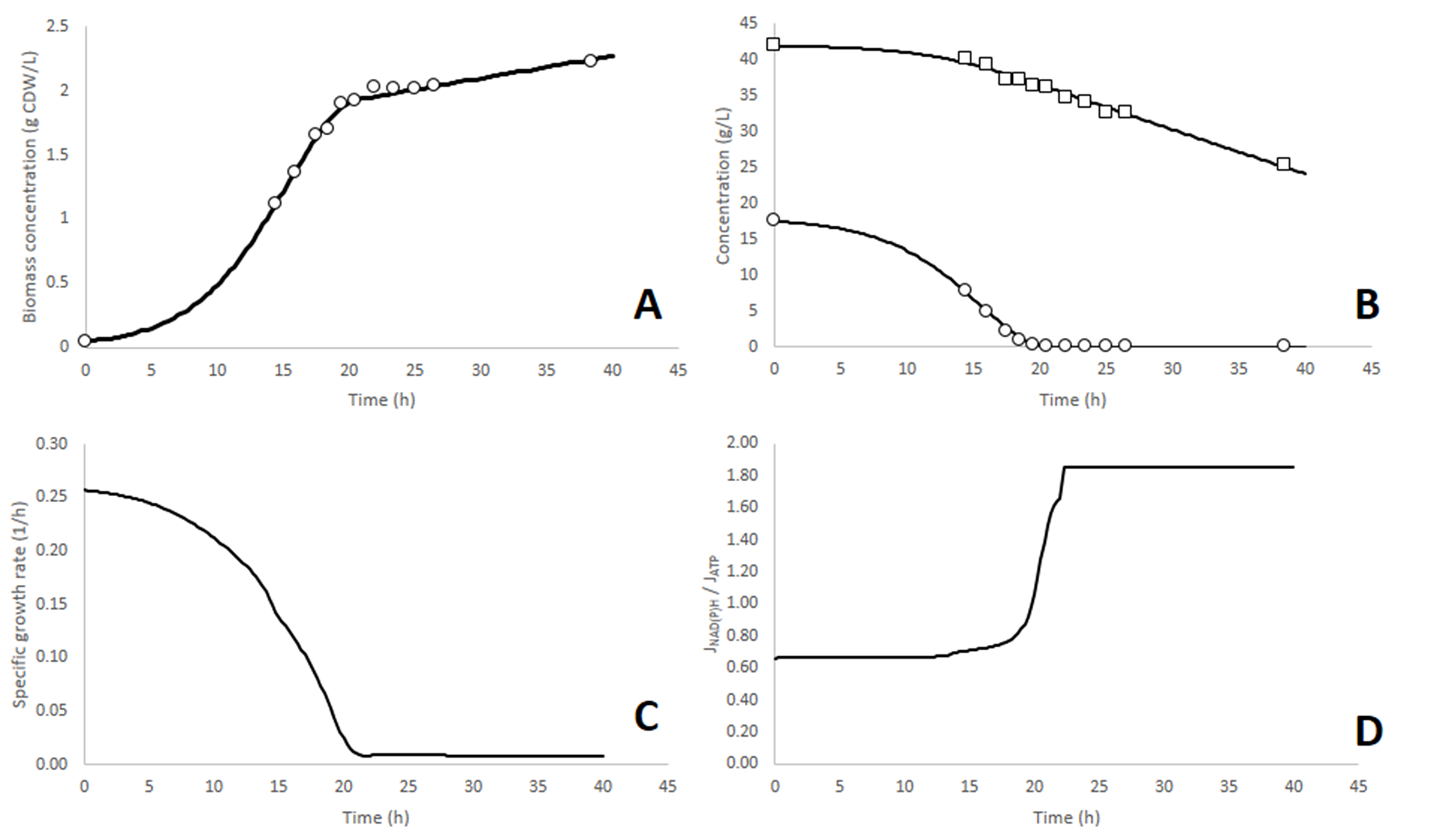

Supplement: Figure S3 — Transition profiles of the metabolism of an anaerobic culture of S. cerevisiae TMB 3057 from glucose to xylose in batch cultures as described in Bergdahl et al. (2012). The symbols represent the measured data points and the lines are generated by the simulation model. (A) Biomass production. (B) Glucose (o) and xylose (□) consumption. (C) Specific growth rate. (D) Change of the cofactor formation flux ratio (RJ) from simultaneous glucose and xylose metabolism to exclusive xylose metabolism. [file Image3.TIF]
