# Supplementary material for: Close to the Edge: Growth Restrained by the NAD(P)H/ATP Formation Flux Ratio
Source: Front Microbiol. 2017 Jun 22;8:1149. doi: 10.3389/fmicb.2017.01149 (PMC5479917; doi:10.3389/fmicb.2017.01149)
Supplement: Table S1 — Metabolite consumption and production rates for S. cerevisiae during exponential growth on xylose. Symbols: μ, specific growth rate (h−1); r, specific consumption/production rates (mmol·g DW−1·h−1); rS, specific substrate consumption rate; rXol, specific xylitol production rate; rG, specific glycerol production rate; rA, specific acetate production rate; rE, specific ethanol production rate. [file Table1.DOCX]

Supplement table S1. Metabolite consumption and production rates for *S. cerevisiae* during exponential growth on xylose. Symbols: µ, specific growth rate (h^-1^); r, specific consumption/production rates (mmol·g DW^-1^·h^-1^); r_S_, specific substrate consumption rate; r_Xol_, specific xylitol production rate; r_G_, specific glycerol production rate; r_A_, specific acetate production rate; r_E_, specific ethanol production rate.

| Strain | r_S_ | r_Xol_ | r_G_ | r_A_ | r_E_ | Reference |
| --- | --- | --- | --- | --- | --- | --- |
| TMB 3001 | 1.37 | 0.75 | 0.031 | 0.04 | 0.85 | Wahlbom & Hahn-Hägerdal, 2002 |
| C1 | 4.00 | 1.26 | 0.25 | 0.08 | 3.81 | Sonderegger et al., 2004 |
| C1 (acetoin) | 4.11 | 0.73 | 0.18 | 0.12 | 4.85 | Sonderegger et al., 2004 |
| TMB 3415 | 2.14 | 0.38 | 0.11 | 0 | 2.63 | Rundquist et al., 2009 |
| TMB 3421 | 3.80 | 0.99 | 0.20 | 0 | 4.35 | Rundquist et al., 2010 |
| TMB 3422 | 4.13 | 0.99 | 0.18 | 0 | 5.00 | Rundquist et al., 2010 |
| TMB 3220 | 5.93 | 0.79 | 0.49 | 0 | 6.96 | Rundquist et al., 2010 |
