# Supplementary material for: Close to the Edge: Growth Restrained by the NAD(P)H/ATP Formation Flux Ratio
Source: Front Microbiol. 2017 Jun 22;8:1149. doi: 10.3389/fmicb.2017.01149 (PMC5479917; doi:10.3389/fmicb.2017.01149)
Supplement: Table S2 — Metabolite consumption and production rates for L. reuteri during exponential growth on glucose or sucrose. Symbols: μ, non-normalized specific growth rate (h−1); r, specific consumption/production rates (mmol·g DW−1·h−1); rS, specific substrate consumption rate; rL, specific lactate production rate; rE, specific ethanol production rate; rA, specific acetate production rate. [file Table2.DOCX]

Supplement table S2. Metabolite consumption and production rates for *L. reuteri* during exponential growth on glucose or sucrose. Symbols: µ, non-normalized specific growth rate (h^-1^); r, specific consumption/production rates (mmol·g DW^-1^·h^-1^); r_S_, specific substrate consumption rate; r_L_, specific lactate production rate; r_E_, specific ethanol production rate; r_A_, specific acetate production rate.

| Strain | µ | substrate | r_S_ | r_L_ | r_E_ | r_A_ | Reference |
| --- | --- | --- | --- | --- | --- | --- | --- |
| ATCC 55730 | 0.54 | Glucose | 0.69 | 0.89 | 0.70 | 0.00 | This study |
| ATCC 55730 | 0.46 | Glucose | 0.82 | 0.85 | 0.72 | 0.00 | This study |
| DSM 17938 | 0.68 | Glucose | 0.84 | 1.18 | 0.12 | 0.39 | This study |
| DSM 17938 | 0.78 | Glucose | 0.95 | 1.11 | 0.78 | 0.00 | This study |
| ATCC 55730 | 0.82 | Sucrose | 0.69 | 0.80 | 0.24 | 0.34 | Årsköld et al 2008 |
