# Supplementary material for: Close to the Edge: Growth Restrained by the NAD(P)H/ATP Formation Flux Ratio
Source: Front Microbiol. 2017 Jun 22;8:1149. doi: 10.3389/fmicb.2017.01149 (PMC5479917; doi:10.3389/fmicb.2017.01149)
Supplement: Table S3 — Parameter values of the kinetic equations to estimate the fermentation profiles of the external fluxes of S. cerevisiae strain 3057. Symbols: Vmaxglc, the maximum specific glucose conversion rate; Kmglc, the affinity constant for glucose; Kixyl, the xylose inhibition constant for glucose conversion; Ki,etohglc, the ethanol inhibition constant for glucose conversion; Vmaxxyl, the maximum specific xylose conversion rate; Kmxyl, the affinity constant for xylose; Kiglc, the glucose inhibition constant for xylose conversion; Ki,etohxyl, the ethanol inhibition constant for xylose conversion; Yglyc/(glc+xyl), the glycerol yield on glucose and xylose; Yglyc/xyl, the glycerol yield on xylose. The ratio between the XR:NADH and XR:NADPH reactions was described by a generalized logistic function with the following parameters: A, the lower asymptote; K, the upper asymptote; Q, affects the value of the ratio at time, t = 0; B, the growth rate; M, sets the starting time; ν, affects near which asymptote maximum growth occurs. [file Table3.DOCX]

Supplement Table S3. Parameter values of the kinetic equations to estimate the fermentation profiles of the external fluxes of S. cerevisiae strain 3057. Symbols: $V_{max}^{glc}$, the maximum specific glucose conversion rate; $K_{m}^{glc}$, the affinity constant for glucose; $K_{i}^{xyl}$, the xylose inhibition constant for glucose conversion; $K_{i,etoh}^{glc}$, the ethanol inhibition constant for glucose conversion; $V_{max}^{xyl}$, the maximum specific xylose conversion rate; $K_{m}^{xyl}$, the affinity constant for xylose; $K_{i}^{glc}$, the glucose inhibition constant for xylose conversion; $K_{i,etoh}^{xyl}$, the ethanol inhibition constant for xylose conversion; Y_glyc/(glc+xyl)_, the glycerol yield on glucose and xylose; Y_glyc/xyl_, the glycerol yield on xylose. The ratio between the XR:NADH and XR:NADPH reactions was described by a generalized logistic function with the following parameters: A, the lower asymptote; K, the upper asymptote; Q, affects the value of the ratio at time t=0; B, the growth rate; M, sets the starting time; ν, affects near which asymptote maximum growth occurs.

| Parameter | Value | Unit |
| --- | --- | --- |
| $V_{\max}^{glc}$ | 17.03 | mmol/g DW/h |
| $K_{m}^{glc}$ | 1.71 | mM |
| $K_{i}^{xyl}$ | 33.34 | mM |
| $K_{i,etoh}^{glc}$ | 181.87 | mM |
| $V_{\max}^{xyl}$ | 7.16 | mmol/g DW/h |
| $K_{m}^{xyl}$ | 129.95 | mM |
| $K_{i}^{glc}$ | 126.96 | mM |
| $K_{i,etoh}^{xyl}$ | 167.48 | mM |
| $Y_{glyc/(glc+xyl)}$ | 0.25 | mmol/mmol |
| $Y_{glyc/xyl}$ | 0.0415 | mmol/mmol |
| *A* | 0.353 |  |
| *K* | 0.950 |  |
| *Q* | 0.0096 |  |
| *B* | 1.940 |  |
| *M* | 11.745 |  |
| *v* | 0.550 |  |
